# Socioeconomic inequality in periconceptional folic acid supplementation in China: a census of 0.9 million women in their first trimester of pregnancy

**DOI:** 10.1186/s12884-017-1618-8

**Published:** 2017-12-16

**Authors:** Min Liu, Jing Chen, Jue Liu, Shikun Zhang, Qiaomei Wang, Haiping Shen, Yiping Zhang

**Affiliations:** 1 Department of Epidemiology and Biostatistics, School of Public Health, Peking University, Beijing, 100191 China; 2 Department of Maternal and Child Health, National Health and Family Planning Commission of the PRC, Beijing, China; 3 Key laboratory of Carcinogenesis and Translational Research (Ministry of Education/Beijing), Peking University Cancer Hospital & Institute, Beijing, China

**Keywords:** Folic acid, Epidemiology, Socioeconomic factors, China

## Abstract

**Background:**

To assess socioeconomic inequality in periconceptional folic acid supplementation in China.

**Methods:**

We used data of periconceptional folic acid (FA) supplementation of rural Chinese women from the National Free Preconception Health Examination Project from 2010 to 2012 and socioeconomic level data from the National Bureau of Statistics. We used logistic models to assess the associations between the prevalence of taking FA and the sociodemographic characteristics of the participants, the couples, and the socioeconomic levels of their region of residence.

**Results:**

Of the 907,720 included women, 682,315 (75.62%) of the women reported taking FA. The prevalence of FA supplementation was significantly higher in participants aged 21–29 (75.87%) than in those women aged 40–49 (68.44%, *p* < 0.01). The prevalence of FA supplementation was significantly higher in the region with the highest Per Capita Gross Regional Product than in the regions with lower Per Capita Gross Regional Product (aOR = 12.20 [95% CI:9.54–15.61]). The higher the per capita net income of farmer households in the region, the higher the prevalence of FA supplementation (aOR = 1.95 [95% CI:1.74–2.18]).

**Conclusions:**

The rate of periconceptional FA supplementation among rural Chinese women has increased with the support of China’s Health System Reform policy. However, socioeconomic disparities in periconceptional folic acid supplementation remain.

## Background

Neural tube defects (NTDs), one of the most common and devastating birth defects, remain a major public health concern in China [1–3]. NTDs have been estimated to affect approximately 0.9 million births every year in China, which include 0.25 million clinically visible cases [1]. NTDs can be effectively prevented with periconceptional folic acid supplementation [4, 5]. A review of periconceptional folic acid supplementation suggested a 72% reduction in risk of development of NTDs and a 68% reduction in risk of recurrence compared with either no intervention, placebo, or micronutrient intake without folic acid [4]. Despite worldwide public health campaigns recommending periconceptional daily supplementation of synthetic folic acid to reduce the risk of NTDs, many women are not following these recommendations [5].

In 2001, the Chinese Ministry of Health and the Chinese Disabled Person Federation released a National Action Plan for Reducing Birth Defects and Disabilities in China for 2002–2010. One of the main targets of the Action Plan was to increase periconceptional folic acid intake for all pregnant women to reduce the prevalence of NTDs in China [6]. In 2009, the Chinese central government launched the Health System Reform Plan to revitalize the provision of public health services [7]. Periconceptional folic acid supplementation in rural Chinese women to prevent birth defects was included in a package of major public health services, and FA would be freely provided for all pregnant rural women nationwide [8]. The Chinese central government invested 6.32 billion renminbi (RMB) in order to provide folic acid supplementation for a total of 45.77 million women of childbearing age in rural areas of China from 2009 to 2013 [9].

The efficacy of providing periconceptional folic acid supplementation to women of childbearing age in preventing NTDs depends on the rate of folic acid intake and their compliance [5]. Some research results have shown that the rate of intake of folic acid was influenced by many factors, such as the woman’s age, years of education, knowledge of folic acid, the economic status of her family, and some other socioeconomic factors [5, 10]. National social economic status, public health policies, and health service fairness and accessibility would be likely to influence the rate of folic acid intake of women [11].

We performed a nationwide census of periconceptional folic acid supplementation for women in their first trimester of pregnancy using data through a follow-up visit program from the National Free Preconception Health Examination Project (NFPHEP) database between 2010 and 2012 [12]. The aim of this study was to evaluate the effectiveness and equity of the national program providing free peri-conceptional folic acid supplementation in China. We designed a study with a large sample size to determine the rate of folic acid intake among rural Chinese women of childbearing age and to analyse the association between the rate of folic acid intake with the individual sociodemographic characteristics of the women, their families and the social economic status of their living areas.

## Methods

### Study design

We performed a nationwide, population-based, cross-sectional study. We used data from a follow-up study of rural women in their first trimester of pregnancy from 220 counties in 31 provinces in China between January 1, 2010, and December 31, 2012, to analyze the prevalence of FA supplementation use. We used the social demographic data of the couples from the National Free Preconception Health Examination Project (NFPHEP) and the social economic data of the region in which the women were living from the National Annals of Statistics of China from 2010 to 2012 to analyze the social demographic and economic determinants of preconception folic acid use.

NFPHEP was launched by the Chinese National Health and Family Planning Commission and Ministry of Finance in 2010 to provide free preconception health examinations for rural married couples who planned to become pregnant within the next 6 months [12]. All couples who had made their conception plan were enrolled by local community staff and were provided a free medical examination together with preconception counselling services. Trained local health workers completed a standardized family health file for the participants using data from the questionnaire survey and the medical examination [13]. These women would be followed up once they were determined to be in their first trimester of pregnancy by the local health workers. The pregnant women would then complete a follow-up questionnaire. These files were then included in a web-based electronic data collection system and sent to the national office. The detailed design, organization, and implementation of this project are described elsewhere [14].

### Participants

The study participants were defined as the women from 220 pilot counties in 31 provinces who had participated in the NFPHEP and who were followed-up during their first trimester of pregnancy from January 1, 2010, to December 31, 2012.

### Folic acid supplement use

In the follow-up questionnaire, the women were asked to report data on their supplemental use of folic acid, the frequency of use, and the time period of use. In this study, we defined folic acid supplement use as taking 1 tablet (0.4 mg) of folic acid daily during the following predefined time periods: (a) at least three months before the last menstrual period to the end of the first trimester, (b) at least one to two months before the last menstrual period to the end of the first trimester, (c) after the last menstrual period to the end of the first trimester, or (d) no use at all. The main outcome variable under study was preconception folic acid supplement use, i.e., any use of folic acid supplements that had started before the last menstrual period (categories (a), (b) and (c) above).

### Potential determinants

Based on the family health file for the participants, we abstracted data on the following relevant determinants of folic acid supplement use: the couples’ age, years of education, ethnicities, occupations, and time at study recruitment.

Four economic indicators were used to evaluate the social economic development level of the 31 provinces in China: Per Capita Gross Regional Product, per capita net income of farmer households, Per Capita Consumption Expenditure of Rural Households and Per Capita Cash Consumption Expenditure of Health Care and Medical Services of Rural Households [15]. (1) Per Capita Gross Regional Product is the indicator of regional economic development, and it is calculated by taking the gross domestic product (GDP) of a region over one year and dividing it by the population of the region. (2) Per capita net income of farmer households reflects the average income level of rural residents in a district. Its calculation is the revenue from all sources in rural households after deducting the expenses incurred divided by the number of family members. (3) Per Capita Consumption Expenditure of Rural Households is equivalent to one year of rural residents’ consumer spending on goods and services divided by the population of the region. (4) Per Capita Cash Consumption Expenditure of Health Care and Medical Services of Rural Households refers to the rural family average per person of all cash payments for health care.

### Procedures

This study included a total of 907,829 rural Chinese women who were followed-up during their first trimester of pregnancy. A total of 5559 rural women were rejected due to a lack of information about folic acid use in their follow up questionnaires. Finally, 902,270 participants were included in this study.

We connected the folic acid use information of the participant in the follow-up questionnaires with the couple’s social demographic data from their family health file.

The data of the four social economic indicators above for the 31 provinces were ranked from lower to higher using the National Annals of Statistics of China during 2010–2012. We divided the data of the four social economic indicators above into the following categories: low, middle, high and higher (Table 1).

**Table 1:** Association between rural Chinese women’s sociodemographic characteristics and folic acid use

	Number of Participants N (%)	Use of folic acid N (%)	Crude odds ratio (95% CI)	Adjusted odds ratio (95% CI)
All	907,720 (100.00)	682,315 (75.62)		
Age (years)				
21–30	359,024 (39.79)	556,948 (75.87)	1.000	1.000
31–39	375,056 (41.57)	119,678 (74.86)	0.95 (0.94,0.96)	1.00 (0.99,1.02)
41–49	126,992 (14.07)	5689 (68.44)	0.69 (0.66,0.72)	0.81 (0.77,0.85)
Years of education				
≤ 6	38,456 (4.26)	29,323 (76.25)	1.000	1.000
7–11	574,818 (63.71)	423,811 (73.73)	0.87 (0.85,0.90)	0.86 (0.83,0.88)
≥ 12	279,261 (30.95)	222,951 (79.84)	1.23 (1.20,1.27)	1.03 (1.00,1.06)
Missing	9735 (1.08)	6230 (64)	0.55 (0.53,0.58)	
Ethnicity				
others	46,488 (5.15)	31,657 (68.1)	1.000	1.000
Han	850,661 (94.28)	647,764 (76.15)	1.50 (1.47,1.53)	1.182 (1.15,1.22)
Missing	5121(0.57)	2894 (56.51)	0.61 (0.57,0.65)	
Occupation				
farmer	674,647 (74.77)	505,465 (74.92)	1.000	1.000
worker	86,484 (9.59)	65,457 (75.69)	1.04 (1.03,1.06)	0.89 (0.87,0.92)
others	125,406 (13.90)	100,770 (80.36)	1.37 (1.35,1.40)	1.02 (1.00,1.05)
Missing	15,733 (1.74)	10,623 (67.52)	0.700 (0.67,0.72)	
Time at study recruitment				
2010	183,893 (20.26)	134,131 (73.58)	1.000	1.000
2011	436,761 (48.11)	329,288 (75.88)	1.13 (1.12,1.14)	1.25 (1.23,1.27)
2012	287,175 (31.63)	218,896 (76.53)	1.17 (1.16,1.19)	1.24 (1.22,1.26)

**Fig. 1:**
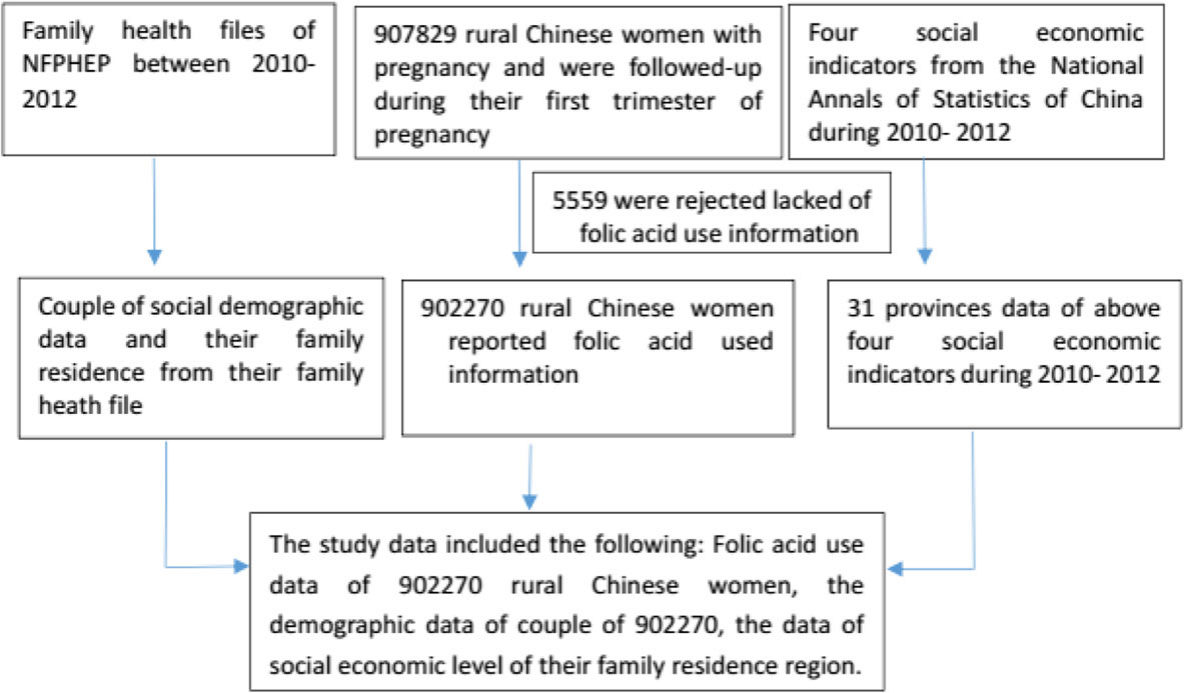
Flowchart of the participants

The recruitment time and information regarding how long the participant had been living in the region were marked and linked to the grade data of the four social economic indicators: the Per Capita Gross Regional Product, the Per capita net income of farmer households, the Per Capita Consumption Expenditure of Rural Households and the Per Capita Cash Consumption Expenditure of Health Care and Medical Services of Rural Households. The participants’ data were summarized in the flowchart in Fig. 1.

### Statistical analysis

All analyses were conducted using SPSS version 18.0 (SPSS Inc., Chicago, IL, USA). The prevalence rate of taking FA was calculated using the participants’ and couples’ sociodemographic information, as well as the socioeconomic level of their families’ region of residence. The association between the prevalence of taking FA and the sociodemographic characteristics of the participants, couples, and the socioeconomic level of their families’ region of residence were also analyzed using the univariate logistic regression model to calculate the crude odds ratio (cOR) with 95% CI. Adjusted odds ratios (aOR) with 95% CIs in the multivariable logistic regression model were calculated after being adjusted for age, region, years of education, occupation, ethnicity, and time at study recruitment of women and their husbands, as well as four socioeconomic indicators of the economic level. Two-sided *p* < 0.05 was considered statistically significant.

## Results

### Prevalence of taking FA by different sociodemographic characteristics of rural Chinese women

**Fig. 2:**
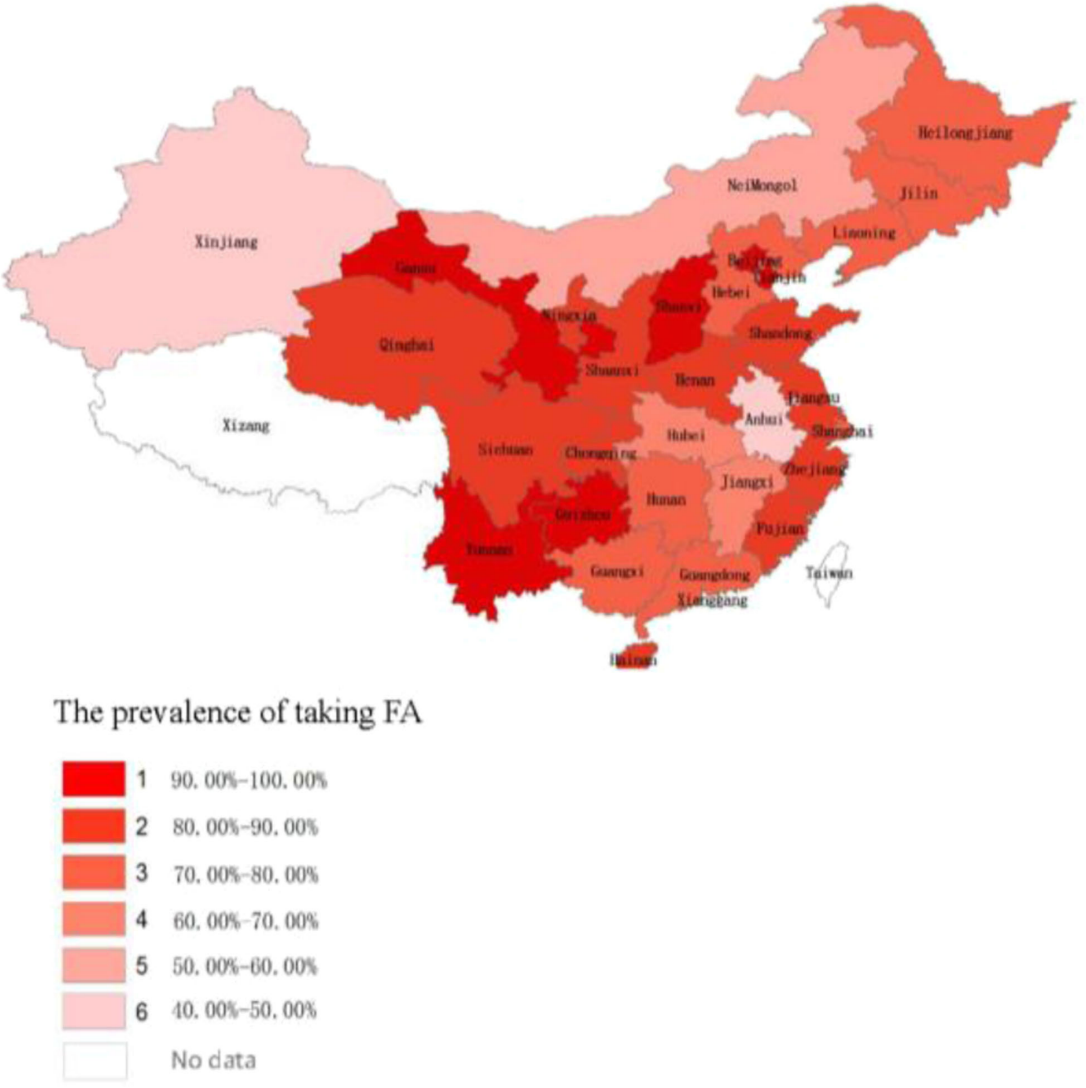
The prevalence of taking FA in different areas

A total of 682,315 participant**s** had reported taking folic acid among the 907,720 women, with an overall prevalence of FA supplementation of 75.62% among the 907,720 rural Chinese women. The FA supplementation prevalence was significantly higher in participants aged 21–29 (75.87%) and 30–39 years (74.86%) than in those aged 40–49 years (68.44%, *p* < 0.001). FA supplementation prevalence was the highest in participants with 12 or more years of education (79.84%), and it was significantly higher than in participants with 6 or fewer years of education (76.25%, *p* < 0.001). Participants of Han ethnicity had a significantly higher prevalence of taking FA than participants of other ethnicities (76.15% vs. 68.105%, *p* < 0.001). Other occupations showed a significantly higher prevalence rate of taking FA than farmers (80.36% vs. 74.92%, p < 0.001). The participants in 2011 and 2012 had a higher prevalence of taking FA than those in 2010 (75.88% and 76.53% vs. 73.58%, *p* < 0.001; Table 1). The prevalence of taking FA in different areas is shown in Fig. 2.

### Prevalence of taking FA by different sociodemographic characteristics of rurual Chinese couples

Among the 902,270 rural couples, the prevalence of FA use varied significantly by age group (*p* < 0.01), and it was significantly higher in couples aged 21–29 years (76.05%) than in other age groups (75.53%–68.23%, *p* < 0.01). The prevalence of FA use varied significantly by years of education; it was significantly higher in couples with 12 years or more years of education (79.96%) than that in couples with 6 or fewer years of education (77.71%, *p* < 0.01). The prevalence of FA supplementation in couples in which either the husband or the wife had received 12 or more years of education was significantly higher than that in less educated couples. Workers or other occupations of the couples was related to a significantly higher prevalence of taking FA than in farmers (75.27% -81.38% vs. 74.72%, p < 0.01) (Table 2).

**Table 2:** Association between rural Chinese couples’ sociodemographic characteristics and folic acid use

Husband	Wife	Number of Couples N (%)	Use of folic acid N (%)	Crude odds ratio (95% CI)	Adjusted odds ratio (95% CI)
Age (years)					
21–29	21–29	525,524 (58.24)	399,650 (76.05)	1.00	1.00
	30–39	19,367 (2.15)	14,609 (75.43)	0.97 (0.94,1.00)	0.97 (0.94,1.01)
	40–49	119 (0.01)	86 (72.27)	0.82 (0.55,1.23)	1.12 (0.73,1.72)
30–39	21–29	158,785 (17.6)	119,595 (75.32)	0.96 (0.95,0.97)	0.98 (0.96,0.99)
	30–39	164,472 (18.23)	124,219 (75.53)	0.97 (0.96,0.99)	1.01 (0.98,1.03)
	40–49	2775 (0.31)	1999 (72.04)	0.81 (0.75,0.88)	0.98 (0.88,1.08)
40–49	21–29	3654 (0.4)	2605 (71.29)	0.78 (0.73,0.84)	0.84 (0.78,0.90)
	30–39	18,367 (2.04)	13,270 (72.25)	0.82 (0.79,0.85)	0.93 (0.89,0.97)
	40–49	9207 (1.02)	6282 (68.23)	0.68 (0.65,0.71)	0.81 (0.74,0.88)
Years of education					
≤6	≤6	17,870 (2)	13,887 (77.71)	1.00	1.00
	7–11	12,487 (1.4)	9417 (75.41)	0.88 (0.83,0.93)	0.91 (0.87,0.95)
	≥12	2712 (0.3)	2075 (76.51)	0.93 (0.85,1.03)	0.89 (0.81,0.97)
7–11	≤6	17,110 (1.92)	12,815 (74.90)	0.86 (0.82,0.90)	0.86 (0.82,0.91)
	7–11	490,464 (55.02)	358,793 (73.15)	0.78 (0.75,0.81)	0.78 (0.77,0.80)
	≥12	49,556 (5.56)	39,394 (79.49)	1.11 (1.07,1.16)	1.02 (1.00,1.05)
≥12	≤6	3412 (0.38)	2572 (75.38)	0.88 (0.81,0.96)	0.88 (0.81,0.96)
	7–11	71,175 (7.98)	55,192 (77.54)	0.99 (0.95,1.03)	–
	≥12	226,641 (25.42)	181,224 (79.96)	1.14 (1.10,1.19)	–
Occupation					
farmer	farmer	640,413 (72.42)	478,538 (74.72)	1.00	1.00
	worker	5633 (0.64)	4125 (73.23)	0.93 (0.87,0.98)	0.71 (0.65,0.77)
	others	9857 (1.11)	7787 (79.00)	1.27 (1.21,1.34)	0.97 (0.92,1.02)
worker	farmer	18,104 (2.05)	14,063 (77.68)	1.18 (1.14,1.22)	1.08 (1.05,1.12)
	worker	72,930 (8.25)	54,896 (75.27)	1.03 (1.01,1.05)	0.78 (0.73,0.82)
	others	19,468 (2.2)	15,575 (80.00)	1.35 (1.31,1.40)	0.98 (0.94,1.02)
others	farmer	14,873 (1.68)	12,044 (80.98)	1.44 (1.38,1.50)	1.29 (1.24,1.35)
	worker	7783 (0.88)	6334 (81.38)	1.48 (1.40,1.57)	–
	others	95,220 (10.77)	76,661 (80.51)	1.40 (1.37,1.42)	–

### Prevalence of taking FA by different socioeconomic status of the region of residence

**Fig. 3:**
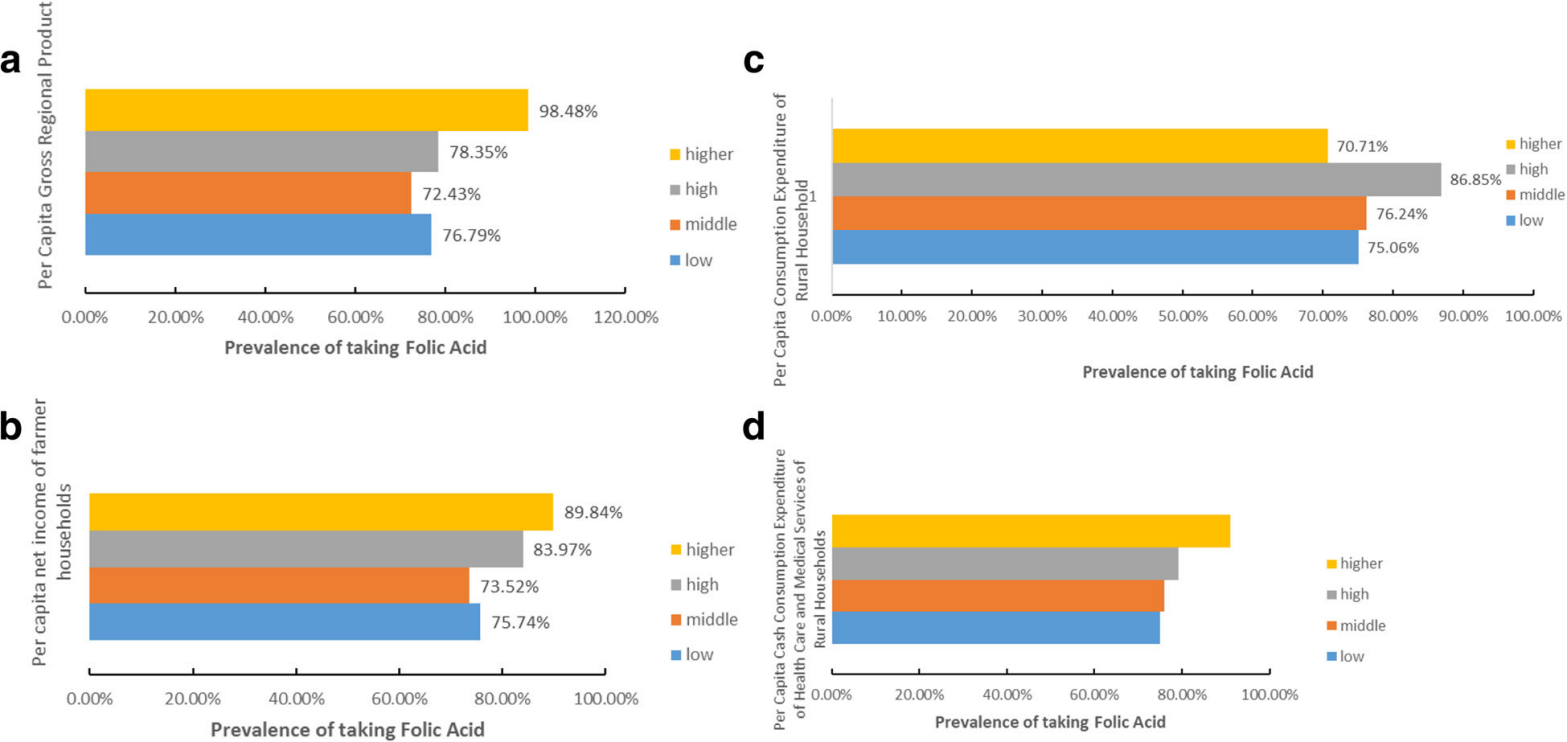
**a** Prevalence of taking folic acid among rural Chinese women living in regions of different Per Capita Gross Regional Product. **b** Prevalence of taking folic acid among rural Chinese women living in regions of different Per Capita net income of farmer households. **c** Prevalence of taking folic acid among rural Chinese women living in regions of different Per Capita Consumption Expenditure of Rural Household. **d** Prevalence of taking folic acid among rural Chinese women living in regions of different Per Capita Cash Consumption Expenditure of Health Care and Medical Services of Rural Households

We analyzed the association between the prevalence of FA supplementation and four indicators of the socioeconomic status of the region (Fig. 3 A-D). When compared with the prevalence of taking FA in regions with lower Per Capita Gross Regional product, the prevalence of taking FA was significantly higher in the region with the highest Per Capita Gross Regional Product (98.48%, aOR = 12.20 [95% CI:9.54–15.61]; Table 3). There was a significant association between the prevalence of FA supplementation and the per capita net income of farmer households. The higher the per capita net income of farmer households in the region, the higher the prevalence of FA supplementation by participants (89.84%, aOR = 1.95[95% CI:1.74–2.18]; 83.97%, aOR = 1.64 [95% CI:1.56–1.72]). The prevalence of FA supplementation was significantly higher in the regions with middle or higher Per Capita Consumption Expenditure of Rural Households compared with the regions with lower Per Capita Consumption Expenditure of Rural Households (76.24%, aOR = 1.07[95% CI:1.04–1.09]; 86.85%, aOR = 1.85[95% CI:1.79–1.90]). There was a significant association between the prevalence of FA supplementation and the Per Capita Cash Consumption Expenditure of Health Care and Medical Services of Rural Households. The higher the Per Capita Cash Consumption Expenditure of Health Care and Medical Services of Rural Households of the region, the higher the prevalence of participants utilizing FA supplementation (74.92%, 75.90%, 79.15%, 91.08%, aOR = 1.05[95% CI:1.03–1.06]; aOR = 1.07 [95% CI:1.04–1.10], aOR = 1.82 [95% CI:1.61–2.05]).

**Table 3:** Association between the socioeconomic levels of rural Chinese women’s region of residence and folic acid use

Economic Indicator	Number of Participants N (%)	Use of folic acid N (%)	Crude odds ratio (95%C.I.)	Adjusted odds ratio (95% CI)
Per Capita Gross Regional Product				
low	492,823 (54.62)	378,439 (76.79)	1.00	1.00
middle	307,224 (34.05)	222,529 (72.43)	0.79 (0.79,0.80)	0.60 (0.60,0.61)
high	95,980 (10.64)	75,199 (78.35)	1.09 (1.08,1.11)	0.67 (0.65,0.69)
higher	6243 (0.69)	6148 (98.48)	19.56 (15.97,23.96)	12.20 (9.54,15.61)
Per capita net income of farmer households				
low	548,559 (60.80)	415,483 (75.74)	1.00	1.00
middle	295,584 (32.76)	217,328 (73.52)	0.89 (0.88,0.90)	1.15 (1.13,1.17)
high	46,316 (5.13)	38,893 (83.97)	1.68 (1.64,1.72)	1.64 (1.56,1.72)
higher	11,811 (1.31)	10,611 (89.84)	2.83 (2.67,3.01)	1.95 (1.74,2.18)
Per Capita Consumption Expenditure of Rural Households				
low	694,268 (76.95)	521,143 (75.06)	1.00	1.00
middle	183,353 (20.32)	139,780 (76.24)	1.07 (1.05,1.08)	1.07 (1.04,1.09)
high	24,550 (2.72)	21,322 (86.85)	2.19 (2.11,2.28)	1.85 (1.79,1.90)
higher	99 (0.01)	70 (70.71)	0.80 (0.52,1.24)	–
Per Capita Cash Consumption Expenditure of Health Care and Medical Services of Rural Households				
low	601,828 (66.70)	450,871 (74.92)	1.00	1.00
middle	231,476 (25.65)	175,694 (75.90)	1.06 (1.04,1.07)	1.05 (1.03,1.06)
high	59,237 (6.57)	46,889 (79.15)	1.27 (1.25,1.30)	1.07 (1.04,1.10)
higher	9729 (1.08)	8861 (91.08)	3.42 (3.19,3.67)	1.82 (1.61,2.05)

### Rates and timing of FA supplementation by different sociodemographic characteristics of rural Chinese women and socioeconomic status of the region of residence

Rates of taking FA at least 3 months before the last menstrual period to the end of the first trimester, at least 1 to 2 months before the last menstrual period to the end of the first trimester and after the last menstrual period to the end of the first trimester were 38.63%, 14.10% and 22.13%, respectively, among 907,720 rural Chinese women.

The rate of FA supplementation for at least 3 months before the last menstrual period to the end of the first trimester was significantly higher in participants aged 31–39 (41.29%), participants with 7–11 years of education (39.4%), participants of Han ethnicity (39.2%) and farmers (39.6%). The rates of FA supplementation for at least 3 months before the last menstrual period to the end of the first trimester in women living in regions with the highest Per Capita Gross Regional product, higher per capita net income of farmer households, the highest Per Capita Consumption Expenditure of Rural Household and Per Capita Cash Consumption Expenditure of Health Care and Medical Services of Rural Households were 82.44%, 48.47%, 68.69% and 46.19%, respectively. There was a significant association between rates of FA supplementation for at least 3 months before the last menstrual period to the end of the first trimester and Per Capita Consumption Expenditure of Rural Households. The regions with higher Per Capita Consumption Expenditure of Rural Households also had higher rates FA supplementation for at least 3 months before the last menstrual period to the end of the first trimester (40.72%, 40.93%, 68.69%, respectively; OR = 1.10[95% CI:1.09–1.11]; OR = 1.11 [95% CI:1.08–1.14], OR = 3.56 [95% CI:2.33–5.45]).

In the univariate logistic regression model, there was an association between the prevalence of FA supplementation with the sociodemographic characteristics of rural Chinese women and the socioeconomic levels of the areas in which their families reside. After adjusting for the participant’s age and their spouse’s age, years of education, ethnicities, occupations, and the social economic levels of the region of residence, the associations still exist (Table 4). There was a higher prevalence of FA supplementation among the younger women, women with more years of education, workers or women with occupations other than farming, and the women living in regions with higher Per Capita Gross Regional Product, per capita net income of farmer households, Per Capita Consumption Expenditure of Rural Households and Per Capita Cash Consumption Expenditure of Health Care and Medical Services of Rural Households.

**Table 4:** Association between rural Chinese women’s sociodemographic characteristics, socioeconomic levels of the region of residence and time period of folic acid use by the multivariable logistic regression model

	Number of Participants (%)	3 months before the last menstrual period to the end of the first trimester		1–2 months before the last menstrual period to the end of the first trimester		After the last menstrual period to the end of the first trimester	
		N (%)	aOR (95% CI)	N (%)	aOR (95% CI)	N (%)	aOR (95% CI)
All	907,720 (100.00)	350,738 (38.63)		130,683 (14.40)		200,894 (22.13)	
Age (years)							
21–30	359,024 (39.79)	281,003 (38.06)	1.000	106,988 (14.49)	1.000	168,957 (22.88)	1.000
21–39	375,056 (41.57)	66,498 (41.29)	1.08 (1.06,1.10)	22,741 (14.12)	1.00 (0.98,1.02)	30,439 (18.90)	0.87 (0.85,0.89)
41–49	126,992 (14.07)	3237 (38.49)	0.89 (0.84,1.00)	954 (11.34)	0.73 (0.67,0.79)	1498 (17.81)	0.71 (0.66,0.76)
Years of education							
≤ 6	38,456 (4.26)	14,813 (38.5)	1.000	6963 (18.11)	1.000	7547 (19.63)	1.000
7–11	574,818 (63.71)	226,632 (39.4)	0.88 (0.85,0.91)	78,859 (13.72)	0.73 (0.70,0.76)	118,320 (20.58)	0.92 (0.89,0.95)
≥ 12	279,261 (30.95)	106,679 (38.2)	0.99 (0.96,1.02)	43,904 (15.72)	0.92 (0.88,0.96)	72,368 (25.91)	1.20 (1.16,1.25)
Missing	9735 (1.08)	2614 (26.9)		957 (9.83)		2659 (27.31)	
Ethic							
others	46,488 (5.15)	16,449 (35.38)	1.000	6878 (14.80)	1.000	8330 (17.92)	1.000
Han	850,661 (94.28)	333,497 (39.2)	1.23 (1.16,1.27)	123,334 (14.50)	1.10 (1.05,1.15)	190,933 (22.45)	1.167 (1.12,1.21)
Missing	5121(0.57)	792 (15.5)		471 (9.14)		1631 (31.84)	
Occupation							
farmer	674,647 (74.77)	267,151 (39.60)	1.00	96,303 (14.27)	1.00	142,011 (21.05)	1.00
worker	86,484 (9.59)	32,617 (37.71)	0.87 (0.84,0.90)	11,748 (13.58)	0.86 (0.83,0.90)	21,092 (24.39)	0.95 (0.92,0.98)
others	125,406 (13.90)	46,375 (36.98)	0.95 (0.93,0.98)	20,767 (16.56)	1.06 (1.02,1.10)	33,628 (26.86)	1.11 (1.07,1.15)
Missing	15,733 (1.74)	4595 (29.21)		1865 (11.85)		4163 (26.46)	
Time at study recruitment							
2010	183,893 (20.26)	71,601 (38.94)	1.00	26,682 (14.51)	1.00	35,848 (19.49)	1.00
2011	436,761 (48.11)	169,415 (38.79)	1.21 (1.20,1.24)	65,325 (15.00)	1.22 (1.19,1.24)	94,548 (21.65)	1.34 (1.32,1.37)
2012	287,175 (31.63)	109,722 (38.21)	1.21 (1.19,1.23)	38,676 (13.47)	1.03 (1.01,1.06)	70,498 (24.55)	1.45 (1.42,1.48)
Per Capita Gross Regional Product							
low	492,823 (54.62)	198,539 (40.29)	1.00	75,028 (15.22)	1.00	104,872 (21.28)	1.00
middle	307,224 (34.05)	108,850 (35.43)	0.53 (0.53,0.54)	40,969 (13.34)	0.57 (0.56,0.58)	72,710 (23.67)	0.76 (0.74,0.77)
high	95,980 (10.64)	38,202 (39.80)	0.59 (0.57,0.61)	14,053 (14.64)	0.62 (0.59,0.64)	22,944 (23.90)	0.865 (0.832,0.9)
higher	6243 (0.69)	5147 (82.44)	20.75 (16.20,26.58)	633 (10.14)	4.15 (3.19,5.40)	368 (5.89)	2.16 (1.65,2.83)
Per capita net income of farmer households							
low	548,559 (60.80)	214,258 (39.06)	1.00	81,937 (14.94)	1.00	119,288 (21.75)	1.00
middle	295,584 (32.76)	110,123 (37.26)	1.176 (1.15,1.201)	39,603 (13.40)	1.22 (1.18,1.25)	67,602 (22.87)	1.08 (1.06,1.11)
high	46,316 (5.13)	22,448 (48.47)	1.58 (1.50,1.67)	5986 (12.92)	1.52 (1.42,1.64)	10,459 (22.58)	1.85 (1.74,1.97)
higher	11,811 (1.31)	3909 (33.10)	1.24 (1.09,1.40)	3157 (26.73)	3.08 (2.67,3.55)	3545 (30.01)	2.51 (2.20,2.85)
Per Capita Consumption Expenditure of Rural Households							
low	694,268 (76.95)	265,965 (38.31)	1.00	102,820 (14.81)	1.00	152,358 (21.95)	1.00
middle	183,353 (20.32)	74,654 (40.72)	1.23 (1.20,1.26)	23,142 (12.62)	0.89 (0.86,0.92)	41,984 (22.90)	0.95 (0.92,0.97)
high	24,550 (2.72)	10,051 (40.94)	1.87 (1.81,1.93)	4720 (19.23)	0.80 (0.73,0.87)	6551 (26.68)	0.91 (0.85,0.99)
higher	99 (0.01)	68 (68.69)	–	1 (1.01)	0.02 (0.02,0.02)	1 (1.01)	–
Per Capita Cash Consumption Expenditure of Health Care and Medical Services of Rural Households							
low	601,828 (66.70)	234,989 (39.05)	1.00	85,937 (14.28)	1.00	129,945 (21.59)	1.00
middle	231,476 (25.65)	85,046 (36.74)	0.98 (0.97,1.00)	33,865 (14.63)	1.12 (1.10,1.15)	56,783 (24.53)	1.12 (1.10,1.14)
high	59,237 (6.57)	27,359 (46.19)	1.32 (1.30,1.37)	8259 (13.94)	1.13 (1.08,1.18)	11,271 (19.03)	0.67 (0.64,0.70)
higher	9729 (1.08)	3344 (34.37)	1.59 (1.40,1.82)	2622 (26.95)	2.17 (1.89,2.49)	2895 (29.76)	1.46 (1.28,1.67)

## Discussion

The periconceptional folic acid supplementation of women is an effective measure to prevent NTDs. The effect of preventing NTDs is influenced by the rate of folic acid intake and the time of initiation of use among women planning pregnancies [5]. The national program proposal for folic acid supplementation suggested that a daily dose of one 0.4 mg folic acid tablet would be used for 3 months prior to their first trimester of pregnancy for the pre-pregnancy women [16]. The researchers considered that there was still a 40–50% decrease in NTDs compared with non-users among women who started folic acid supplementation during the first trimester or after the last menstrual period [17].

Tort J’s study results showed that 14.8% (95% CI, 14.2–15.4) of women used folic acid before pregnancy; this percentage varied from 10.4% to 18.7% across regions in France, 2010 [18]. In one Italian survey performed in 2012, a total of 500 women were surveyed: 217 (43.4%) took folic acid before becoming pregnant, and 283 (56.6%) did not take it [19]. McKeating A et al. analyzed trends in folic acid supplementation among women scheduling antenatal care between 2009 and 2013 in Europe [20]. Overall, 43.9% of the women (*n* = 18,473) took periconceptional (preconceptional and postconceptional) folic acid, 49.4% (*n* = 20,782) took postconceptional folic acid only, and 6.6% (*n* = 2787) took no folic acid. The periconceptional folic acid rate decreased from 45.1% in 2009 to 43.1% in 2013. Preconception folic acid use was reported by 23.5% (*n* = 515) of the participants from seven maternity clinics located in six Italian regions. Of these participants, 479 (93%) women had taken folic acid supplements on a daily basis as recommended by the health authorities [21]. In China, the data of some studies with small sample sizes had shown that preconceptional and postconceptional folic acid use rates were 10.2% (2003–2005), 14.9% (2008), and 67.7% (2008–2009) in Shanxi, Shanghai and Anhui during 2005–2008 [22–24]. Our finding of the rates of folic acid supplementation among women in their first trimester of pregnancy were 73.58% (2010), 75.86% (2011) and 76.53% (2012) respectively, from 2010 to 2012. Of these women who took FA, 38.63%, 14.40% and 22.13% of the women had begun FA supplementation in the 3 months before the last menstrual cycle, 1–2 months before the last menstrual cycle and after the last menstrual cycle, respectively. Our findings showed that the prevalence of periconceptional folic acid supplementation among rural Chinese women was apparently higher than the findings of the above studies from Europe and other countries, and it was also higher than the findings of some studies with small sample sizes from China.

In the national management plan for the program on folic acid supplementation to prevent neural tube defects in 2010, the Chinese government proposed at least 70% and 90% of all women who marry and plan to become pregnant would consume 400 μg/day before conception through the end of the first trimester by the end of 2010 and 2011 [16]. Our findings showed that the annual targets of 70% folic acid supplementation rates in 2010 had been reached, but these targets were not met in 2011 among rural Chinese women. Our study also found that approximately 60% of women were not taking folic acid supplementation in the 3 months before the last menstrual period, which is the time thought to be most effective for preventing NDTs. It is very important for controlling birth defects that the rate of FA supplementation in women is increasing, especially the proportion of women taking folic acid before their pregnancy.

Studies have shown that women’s social demographic characteristics could be associated with the rate of periconceptional folic acid supplementation. The lower rates of preconception folic acid use were associated with some characteristics of the women such as older age, fewer years of education, ethnic minorities and women who were unemployed [4, 10, 11, 21]. Our findings showed that the age, years of education, ethnicity and occupation of the women were important determinants of periconceptional folic acid use among rural Chinese women. These results were consistent with those of other studies [18, 21]. To improve the rate of folic acid use, it is important to educate and spread information about the periconceptional use of folic acid, especially for older, less educated women living in rural area.

The association with the social demographic characteristics of couples preparing for pregnancy and the rate of periconceptional folic acid supplementation was analyzed in our study. Our findings showed that there were some lower rates of folic acid supplementation among these women if their husbands were older, were less educated and worked as a farmer. However, the higher FA supplementation rates were found among the younger couples, those with more years of education, and couples who were professional workers. Our findings demonstrated that the periconceptional folic acid supplementation of women was also influenced by their husband’s characteristics. Increasing the couple’s knowledge of folic acid supplementation to prevent birth defects also may improve women’s compliance and the prevalence of periconceptional folic acid supplementation.

The socioeconomic status of the country could importantly influence the implementation effect of the public health project [5, 25–27]. In China, the social economic status of different regions directly affected the provision and utilization of public health services [3, 6]. We analyzed an association with the rates of periconceptional folic acid supplementation and four socioeconomic indicators of Per Capita Gross Regional Product, Per capita net income of farmer households, Per Capita Consumption Expenditure of Rural Household and Per Capita Cash Consumption Expenditure of Health Care and Medical Services of Rural Households. Our finding indicated that the folic acid supplementation rate of women was associated with the above four socioeconomic indicators, of which the Per capita net income of rural households and Rural residents per capita consumption expenditure were especially associated. There were higher rates of FA supplementation among the women living in the regions with higher levels of Per capita net income of rural households and Rural residents per capita consumption expenditure. The FA supplementation rate was still associated with socioeconomic indicators after controlling women’s individual factors and their family factors.

From the Health System Reform Plan in China 2009, the periconceptional folic acid supplementation for rural women preparing for pregnancy had been one of the major public health service projects provided by the Chinese government. The Chinese government not only purchased the folic acid tablets to provide free for the rural women, but they also offered some subsidies to motivate village doctors to provide this service [7, 8]. Our study found lower FA supplementation rates among women living in regions with lower levels of socioeconomic status. This finding indicated that the women living in the regions with less-developed economies and/or in families with poor-economic conditions may still find it difficult to obtain these services, although the folic acid pills and a reward for the primary medical staff were provided by the government. Our study suggests that the government should give more preferential policies for these women living in the regions with less-developed economies and/or in families with poor economic condition in order to improve the accessibility of public health services and promote the realization of equalization.

Our study has some limitations. First, we used data from the NFPHEP database from January 1, 2010, to December 31, 2012, of a total of 902,270 women who followed-up during their first trimester of pregnancy from 220 pilot counties of 31 provinces. The number of women who took FA, but who were not pregnant from January 1, 2010, to December 31, 2012, were not included. Therefore, it is possible that the FA supplementation rate in our study may be lower than the actual rate of FA supplementation. Second, we would classify China’s 31 provinces by the four levels according to four social economic indicators and described the difference of FA supplementation rates among women living in different social economic status regions. There were the relatively small numbers of women in the best social economic regions of China in our data. The FA supplementation rate of women living in these regions may be underestimated because the proportion of rural residents is low in the economically developed regions. Third, the representativeness of the per capita income in a country similar to China, with a high Gini index, may not be the best way to compare the relationship between folic acid use and socioeconomic level.

The main strengths of our study lie in the largest sample study of periconceptional folic acid supplementation for childbearing age women of in China that recruited all women in their first trimester of pregnancy from 220 counties of China between 2010 and 2012. The FA supplementation data of this study came from the self-reports of women when they were followed-up in their first trimester of pregnancy. Compared with other studies [19, 22, 26], our research subjects were taking folic acid in their first trimester of pregnancy. Therefore, our results may have less recall bias. Our results can not only provide rural Chinese women of childbearing age the latest data of folic acid supplementation in pregnancy but also show the rate difference in the rate of supplementation with folic acid among the rural Chinese women with different demographic characteristics and those living in regions with a different socioeconomic status. Our study may also provide evidence for some related policies in order to achieve the target for public health service equalization in China.

## Conclusion

In conclusion, the rate of periconceptional folic acid supplementation among rural Chinese women is higher with the support of China’s Health System Reform policy. However, the absolute number of rural Chinese women who did not use folic acid is very large and the socioeconomic and demographic disparities in the rate of periconceptional folic acid supplementation of rural Chinese women are obvious.

## Abbreviations

aOR: Adjusted odds ratio
CIs: Confidence intervals
cOR: Crude odds ratio
FA: Folic acid
NTD: Neural tube defects
